# Genetics and genomic medicine in Slovakia

**DOI:** 10.1002/mgg3.122

**Published:** 2015-01-08

**Authors:** Ludevít Kádaši, František Cisárik

**Affiliations:** 1 Institute of Molecular Physiology and Genetics, Slovak Academy of SciencesBratislava, Slovakia; 2 Faculty of Natural Sciences, Comenius UniversityBratislava, Slovakia; 3 Department of Medical Genetics, Faculty HospitalŽilina, Slovakia

## Introduction

The Slovak Republic (SR) is a small sovereign state in Central Europe which gained independence on the 1 January 1993 by separation from the Czech/Slovak Federal Republic. From 2002, the SR was divided into eight semi-autonomous Regions (“*krajov*”); each named after its principal city; with their self-governing bodies referred to as Upper-Tier Territorial Units (VÚC). These regions are subdivided„ into a total of 79 districts (“*okresov*”). The Slovak Republic joined the European Union on 1st January 2004 and was included in the Eurozone on 1 January 2009.

SR land area covers 49,036 square kilometers with a 2011 population of 5,397,036 (http://www.portal.statistics.sk) in three main ethnic groups: 80.7% Slovaks, 8.5% Hungarians, and 2% Romany. The Roma percentage is actually greater than this; estimated between 7.5% and 8.8%, as many Roma declared their census ethnicity Slovak or Hungarian dependent on their habitat, and a further 380,000 appear not to have furnished 2011 census particulars. Romany presence on Slovak soil was first recorded in 1322 AD, and significant migration waves were later reported in the 16–17th centuries from Western Europe, and again in the second half of 19th century from Vlachia and Moldavia after slavery's abolition. Romany people currently inhabit ∼300 SR settlements.

While Slovak and Hungarian families prevailingly have two children, and rarely three or more, Roma families have a significantly higher number; often ten or more.

## General Health Services

The right of all SR citizens to health care is constitutionally guaranteed; with general health services regulated by the following main 2004 laws: (1) Law 577/2004 regulates the extent of healthcare financed from public health insurance, and (2) Law 578/2004 defines the public network of healthcare providers, the licensing conditions for founding healthcare facilities. Personnel qualification requirements and continued education, and a quality control system overseeing healthcare duties of both provider personnel and professional associations. These laws have been updated several times; with healthcare service management delegated to the Ministry of Health (MH) and the Bureau for Supervision of Healthcare Provision, and the institution of healthcare facilities founded by both state and private corporations.

SR healthcare service expenses are financed from public health insurance based on agreement between healthcare providers and insurance companies. There are currently three SR insurance companies: one state founded and two private companies. All employed Slovak inhabitants are obliged to pay a legally assigned health insurance fee from their income, freely choosing their contracted insurance company; while the state pays for children, students, and pensioner groups. Healthcare services financed by individual insurance companies are substantially similar, with exceptional services negotiated with providers for patient payment, and those unavailable in the SR can be accessed internationally with insurance company negotiation and consent.

## Genetic Services and Testing

### Legislative limits

Although the Slovak republic has no specialized healthcare laws regulating the provision of genetic services over and above the quoted 577/2014 and 278/214 laws, generalized rules are implemented under Health Ministry powers and guidelines The most important of these is titled “The *Conceptual Framework of Medical Genetics*.” This was originally issued in 1972; with its most recent amendment in 2013 defining the following: (1) the content and extent of medical genetics services, (2) the criteria for building the insurance provider network, (3) approved healthcare facility types with infrastructure and personnel requirements for their creation and function, and (4) a quality control system ensuring ongoing personnel education.

A further regulation level in genetics services provision is overseen by the *Chief Specialist for Medical Genetics* appointed by the Ministry of Health. The main duties include regulating interaction between healthcare service providers and the MH, cooperating with MH issued regulations which demand a high professional level of genetic services provision and supervising regulation adherence.

Artificial pregnancy termination in SR is regulated by law no. 73/1986, which allows interruption without providing a reason until the 12th gestational week, while serious fetal pathology or endangering the mother's life allows pregnancy termination until the 24th gestational week. Maternal informed consent is required for all terminations.

Medical genetics legislation empowered in 1964 demands compulsory reporting of congenital abnormalities Here, data collection and evaluation are performed by the National Health Information Centre (NHIC, http://www.nczisk.sk). This mandated reporting of genetic disorders was instituted in 2014 and the Slovak Republic Genetic Registry created. NHIC also conducts the following registries; the National Registry for Congenital Heart Defects (established in 1992), the National Cancer Registry – including rare cancers – (established in 1976), and the National Child Diabetes Mellitus and Neonatal Diabetes Registry (established in 1986).

Current European Union (EU) healthcare provisions demand complex management of patients with rare diseases. In compliance, the SR Government passed its 2012 resolution entitled “National strategy for improvement of healthcare provided to patients affected with rare disorders for years 2012–2013;” whereby Health ministry experts instituted the “National plan for improvement of healthcare provided to patients affected with rare disorders in SR.” It is estimated that ∼80% of rare disorders are genetic in origin.

### Genetics professionals

Slovak facilities providing medical genetics services employ the following graduate professionals; (1) physicians specializing in clinical medical genetics and (2) nonmedical university graduates in genetics, biochemistry, microbiology, and molecular biology.

*Medical genetics* has been an acknowledged medical speciality in Slovakia since 1972. The undergraduate curriculum in all medical faculties includes general genetics; with medical genetics clinical applications broached in individual clinical branches. The main medical genetic specialist education is conducted at the postgraduate level over 4 years and is completed by passing board examinations and defending a written dissertation. NHIC 2012 data indicate 39 physician specialists in medical genetics in 23 facilities; which approximates 1one specialist for 138,000 inhabitants. In contrast, nonphysician professional postgraduate education lasts 3 years and postgraduates achieve specialization in “*analytical methods in medical genetics*” by passing board examinations and defence of a written dissertation. Although these medical genetics specialists outnumber physicians, no precise data are available. In addition to graduate professionals, personnel with secondary school education, nurses, and laboratory technicians are employed at facilities providing medical genetics services. These employees access education systems to achieve speciality in their respective genetic services fields.

*The Slovak Society of Medical Genetics (SSMG)* is a professional association for all medical genetics personnel and for professionals in alternate medical fields interested in medical genetics. SSMG has a common history with the Czech Society of Medical Genetics. It began here in 1967, and although the Czech and Slovak medical genetic societies separated in conjunction with 1993 statehood, they maintain close collaboration. Current SSMG membership varies from 110 to 120; with the main purpose of promoting member's professional skills and their activity in organized workshops, seminars, and congresses. The initial congress was organized in 1974, and this annual event was then renamed “The Izakovič's Memorial” in 1990 to honor Professor Viliam Izakovič; founder and outstanding expert in Slovak medical genetics. This annual memorial congress encompasses all fields of medical genetics and it currently alternates between SR cities, with an average 100 oral and poster presentations.

### Organization of medical genetics services providers

Medical genetics in SR forms an integral part of the healthcare system and according to the *Conceptual Framework of Medical Genetics,* these services have three types of listed providers; (1) medical genetics departments, (2) medical genetics outpatient clinics and (3) specialized laboratories.

1. Departments: medical genetics university and faculty hospitals departments are involved in outpatient clinics, specialized laboratories and perform research.
2. Outpatient clinics: these were founded in the SR at the beginning of the 1970s, and the current dense network throughout Slovakia is illustrated on our map (Fig.1). These are often the first facility contact for patients, ensuring accessibility to genetic services in all national regions. Patients either attend the clinics voluntarily or are referred by medical specialists. The clinics’ major task is initial overall patient management of genetic disease, including identifying genealogy and the indications for specific testing and genetic counseling. The majority of outpatient clinics perform basic cytogenetic analysis and some also conduct molecular genetic analysis. Testing of samples outside the scope of individual clinics and their attendant laboratories is performed in relevant Slovak facilities, and patients requiring international testing must have prior health insurance company approval. Most importantly, patient or legal representative informed consent is compulsory for all genetic testing.

**Figure 1:**
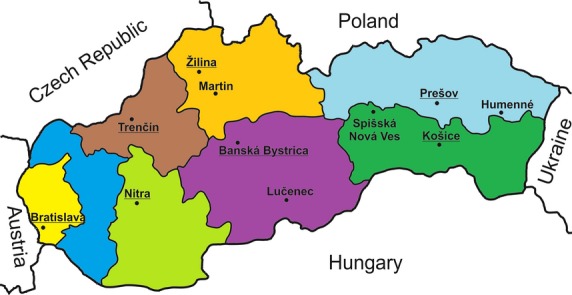
Map of Slovakia showing distribution of medical genetic services providers. The principal city of the respective Region is underlined.
3. Specialized laboratories; these perform genetic testing on the DNA molecular level. There are currently three SR laboratories specializing in onco-genetics and three in monogenic DNA diagnostics. Sample testing here is financed by public health insurance following specialist genetic physician referral.

Specialized diagnostic centers, especially in university and faculty hospitals, have been created for frequently occurring genetic disorders. These supply complex management for patients from wide regional and national areas; with three centers for cystic fibrosis, two for phenylketonuria, and one each for hemophilia, alkaptonuria, muscular dystrophies, neurofibromatosis, and rare metabolic disorders. In addition, patients with monogenic forms of cancers are treated in SR oncologic clinics, where karyotyping, FISH and molecular genetics testing are available.

Medical genetic service providers are either state-owned or private companies which meet law no. 578/2004 criteria and conform to the *Conceptual Framework of Medical Genetics*. These services are financed from public health insurance by agreement between provider and insurance company; with patients bearing the cost of unreferred and unlegislated care. While in SR genetic testing is also offered by domestic and foreign companies via “direct-to-consumer” testing, no precise data on its prevalence are available.

## Genetic Testing Cover Under the Public Healthcare System

The availability of publicly funded diagnostic genetic testing follows a similar distribution pattern to that for general public medical services.

Cytogenetic traditional karyotyping and FISH testing is widely available for both clinical and prenatal diagnosis. Molecular karyotyping (qPCR) is mostly available only in private laboratories, where chromosomal microarray analysis is offered by two laboratories with different platforms, and cytogenetics for oncology and onco-hematology is performed in specialized oncology clinic laboratories.

Initial diagnostic DNA molecular testing in SR was performed in 1987 for prenatal diagnosis of cystic fibrosis by linkage to DNA polymorphisms. The scale of genetic disorders and molecular techniques have significantly expanded, so that current diagnostic testing is amenable for more than 300 monogenic disorders; covering all frequent disorders and a relatively large number of rare ones. While limited insurance-approved and patient-funded testing is available for Slovak patients internationally. In SR diagnostic molecular testing for alkaptonuria is so well established because of its high incidence that worldwide samples are tested in our laboratories.

A broad scale of progressive molecular genetics techniques are employed, including: PCR (Polymerase Chain Reaction), MLPA (Multiple Ligation-dependent Probe Amplification) and fragment analysis modifications, Sanger's sequencing, expression analysis, micro deletion/duplication and CNV microarrays; with massive parallel sequencing (NGS) also available to a limited extent. These tests are mostly offered by private providers, because running these facilities is very expensive and the majority of state-funded healthcare providers are deeply in debt. Hence, finance-shortage constitutes the main limiting for molecular genetic testing factor in SR. Insurance company budgets assigned to genetic testing are well below the medically justified demand, ensuring that waiting-time for some tests ranges from several months to a year.

### Prenatal diagnosis

Prenatal diagnosis has been widely available in Slovakia since initial karyotyping in the mid 1970s. Prenatal testing must be indicated by a medical genetic specialist based on established and unacceptable fetal risk, family history, molecular testing, or biochemical and ultrasonography screening. Genetic counseling is mandatory, both before and after prenatal testing; with all incurred expenses covered by public health insurance.

Samples obtained from amniocentesis or CVS (Chorionic Villus Sampling) are typically used in karotyping chromosomal analysis. Rapid interphase fluorescence in situ hybridization (FISH) screening for chromosomal aneuploidy, evaluation for specific microdeletions or microduplications, microarray and other innovative molecular techniques are also available.

Although some private fertility clinics offer preimplantation genetic testing, they send their samples abroad because test costs are not covered by SR public health insurance.

## National Screening Program

### Neonatal screening

Neonatal screening policy in SR (NBS) was officially established by the Ministry of Health in 1985. Its latest modification was in 2012, increasing the number of screened disorders to the 14 listed in Table1 (Statute 42, Bulletin of MH SR, parts 39–60). Screening in Slovakia is provided by the National Newborn Screening Centre (NNSC) in Banská Bystrica; coordinated with three Regional Recall Centres; and NNSC is a functioning member of EUNENBS (European Union Network of Experts on Newborn Screening).

**Table 1:** Currently screened disorders in SR.

No.	Disorder	Abbreviation	Screening begun
1	Congenital hypothyroidism	CH	1985
2	Congenital adrenal hyperplasia	CAH	2003
3	Cystic fibrosis	CF	2009
4	Phenylketonuria	PKU	1985
5	Hyperphenylalaninemia	HPA	2013
6	Maple syrup urine disease	MSUD	2013
7	Deficiency of acyl-CoA-dehydrogenase for medium-chain fatty acids	MCAD	2013
8	Deficiency of 3-hydroxyacyl-CoA-dehydrogenase for long-chain fatty acids	LCHAD	2013
9	Deficiency of acyl-CoA-dehydrogenase for very long-chain fatty acids	VLCAD	2013
10	Deficiency of carnitínpalmitoyltransferase I	CPT I	2013
11	Deficiency of carnitínpalmitoyltransferase II	CPT II	2013
12	Deficiency of carnitine acylcarnitine translocase		2013
13	Glutarate aciduria, type I	GA I	2013
14	Isovaleric acidemia	IVA	2013

In addition to the listed diseases, every newborn is screened for hearing impairment and hip dysplasia; with over 90% further screened immediately after birth by ultrasonography for somatic CNS (Central Nervous System) malformations, cardiovascular system anomalies, and obstructive uropathy. These latter screenings, however, do not form part of official governmental policy.

### Screening during pregnancy

Gynecologists are obliged to offer every pregnant woman biochemical and ultrasonograpic screening for developmental anomalies; but acceptance is voluntary. The most frequently used approach is combined first trimester screening for pregnancy-associated plasma protein-A (PAPP-A); and free beta human chorionic gonadotropin (hCG) or total hCG with or without nuchal translucency (NT). Integrated screening with or without NT for total human chorionic gonadotropin, maternal serum alpha-fetoprotein and unconjugated oestriol is also conducted.

The increasing quality of biochemical and ultrasonographic screening is well documented by statistics. While 1991 prenatal karyotyping from biochemical and ultrasonographic analyses based on estimated increased risk comprised ∼30% of all prenatal tests, this increased to 61% in 2013. Similarly, the 2002 average of 2.1% identified prenatal chromosomal aberrations increased to 5.1% in 2013.

Prenatal testing acceptance ranges from 80% to 90% in SR large towns, and 50–60% in rural regions. This difference is attributed to possible greater religious ideology and relatively less biology and genetics knowledge in rural communities.

Pregnant Slovak woman over 35 years are legally compelled to undergo prenatal screening, regardless of anticipated outcomes.

## Genetic Burden of the Slovak Population

### Monogenic disorders

The SR non-Romany population exhibits a similar scale and incidence in the majority of genetic disorder as other European populations. The most prevalent monogenic disorders include cystic fibrosis, phenylketonuria, hemophilia A and B, spinal muscular atrophies, Huntington's chorea, Duchenne and Becker muscular dystrophy, myotonic dystrophy, monogenic hearing loss, neurofibromatosis type 1, FRAXA, Wilson's disease, Charcot-Marie-Tooth syndrome, etc.

In contrast, SR alkaptonuria prevalence is an exception, with the second highest world incidence of 1 in 19,000 inhabitants. This compares unfavorably with the worldwide average of ∼1 in 250,000: and hence specialized alkaptonuria research and health care has been paramount. Slovak researchers have played a significant role in elucidating alkaptonuria genetics and instituting treatment (Zaťková et al. 2000a,b). Here, the Piešťany spa, under the auspices of the National Institute for Rheumatic Disorders, is well renowned for its specialized health-team alleviating affected patients’ rheumatic symptoms.

The Slovak Romany form a typical small genetically isolated population, with almost 100% endogamy, a high inbreeding ratio and all characteristics encountered in such populations. It has vast monogenic disorder frequency compared to its non-Romany counterparts. Examples include: (1) Romany suffer one of the highest phenylketonuria incidences in the world; at 1 in 1000 newborn; ten times the frequency registered in the majority SR population. This is caused by founder mutation R252W (Feráková et al. 1992); (2) primary congenital glaucoma is present in 1 in 1200 of their newborn; caused by founder mutation E387K in the *CYP1B1* gene (Plášilová et al. 1998, 1999); (3) monogenic hearing impairments are very frequent; with mutations in *GJB2* and *MARVELD2* genes identified in affected patients. While founder mutation IVS4+2T-C is identified in the *MARVELD2* gene (Šoltýsová et al. 2012), the scale of mutations in the *GJB2* gene is broader with mutation W24X dominating (Minárik et al. 2003); and (4) the recently identified retinitis pigmentosa type caused by founder mutation c. 316C>T in the *RDH12* gene is noted (Ficek et al. 2003).

In direct contrast, cystic fibrosis, one of the most frequent monogenic diseases in European populations, is currently undetected in the Romany population.

### Reporting birth defects

The National Health Information Centre (NHIC) holds most national health registries. Its National Registry for Congenital Disorders established in 2011 from the former Register of Congenital Anomalies in Newborns (1964), highlights the significant proportion of congenital anomalies of genetic origin (http://www.nczisk.sk). This records that 1953 of the total 55,585 SK newborns in 2012 had diagnosed congenital anomalies. Table2 hereunder lists the distribution of these congenital anomalies by type and specification. The parents of these children are referred to local outpatient clinics for medical genetics services and family planning advice.

**Table 2:** Congenital anomalies by type and specification diagnosed in SR in 2012 (*Health Statistics Yearbook of the Slovak Republic* 2012).

Specification according to MKCH-10	Number
Circulatory system (Q20–Q28)	594
Musculoskeletal system (Q65–Q90)	373
Genital organs (Q50–Q56)	352
Urinary system (Q60–Q64)	268
Other congenital malformations (Q80–Q89)	147
Nervous system (Q00–Q07)	92
Cleft lip and cleft palate (Q35–Q37)	92
Other congenital malformations of the digestive system (Q38–Q45)	80
Eye, ear, face, and neck (Q10–Q18)	78
Chromosomal abnormalities (Q99–Q99)	60
Congenital hypothyroidism (E03)	26
Respiratory system (Q30–Q34)	25
Metabolic disorders (E70–E90)	15
Number of children with CA	1953

## Research in Medical Genetics

Medical genetics research in SR is primarily conducted in state institutions; with private company participation almost negligible. Most research is concentrated in Slovak Academy of Sciences entities which include, amongst others: (1) The Institute of Molecular Physiology and Genetics focuses on monogenic disorders; (2) The Institute of Experimental Endocrinology focused on metabolic disorders; and (3) The Institute of Experimental Oncology researches monogenic oncologic disorders. Significant research covering practically all fields of medical genetics is promoted in medical universities and university hospitals. Progressive technological equipment from EU Structural Fund projects is well established, including state-of-the-art massive parallel sequencing devices. The main factor limiting research effectiveness is insufficient finance allocated by research agencies to run this equipment. The two state grant agencies in SR are VEGA (a joint research agency of the Ministry of Education and Slovak Academy of Sciences for institution from these two resorts), and RAPRD (Research Agency for Promoting Research and Development). These agencies cover grants to all SR institutions; both state and private.

## Final Remarks

Provision of medical genetics services in the Slovak Republic has a well-established organizational structure, network of providers, personnel and methodical conditions which ensure high-level services. The main limiting factor is finance allocated to these services by insurance companies; far below eligible demand. Consequently, test waiting-time ranges from several months to a year, with similar protracted introduction of the latest technology and tests in diagnostic practice. These latter are mostly developed and offered by private providers; often via “direct-to-consumer” testing, because state providers are deeply in debt. Medical genetics intervenes in all current and future fields of medicine and its impact can only increase exponentially. Therefore, if the Slovak Republic wishes to emulate well-developed states, our insurance companies must re-evaluate their medical genetics services finance policies.

## Conflict of Interest

None declared.
